# Risk Factors for Patient–Ventilator Asynchrony and Its Impact on Clinical Outcomes: Analytics Based on Deep Learning Algorithm

**DOI:** 10.3389/fmed.2020.597406

**Published:** 2020-11-25

**Authors:** Huiqing Ge, Kailiang Duan, Jimei Wang, Liuqing Jiang, Lingwei Zhang, Yuhan Zhou, Luping Fang, Leo M. A. Heunks, Qing Pan, Zhongheng Zhang

**Affiliations:** ^1^Department of Respiratory Care, Sir Run Run Shaw Hospital, Zhejiang University School of Medicine, Hangzhou, China; ^2^Regional Medical Center for National Institute of Respiratory Diseases, Bethesda, MD, United States; ^3^College of Information Engineering, Zhejiang University of Technology, Hangzhou, China; ^4^Department of Intensive Care Medicine, Amsterdam UMC, Amsterdam, Netherlands; ^5^Department of Emergency Medicine, Sir Run Run Shaw Hospital, Zhejiang University School of Medicine, Hangzhou, China

**Keywords:** patient ventilator asynchrony, mortality, deep learning, mechanical ventilalion, critical care

## Abstract

**Background and objectives:** Patient–ventilator asynchronies (PVAs) are common in mechanically ventilated patients. However, the epidemiology of PVAs and its impact on clinical outcome remains controversial. The current study aims to evaluate the epidemiology and risk factors of PVAs and their impact on clinical outcomes using big data analytics.

**Methods:** The study was conducted in a tertiary care hospital; all patients with mechanical ventilation from June to December 2019 were included for analysis. Negative binomial regression and distributed lag non-linear models (DLNM) were used to explore risk factors for PVAs. PVAs were included as a time-varying covariate into Cox regression models to investigate its influence on the hazard of mortality and ventilator-associated events (VAEs).

**Results:** A total of 146 patients involving 50,124 h and 51,451,138 respiratory cycles were analyzed. The overall mortality rate was 15.6%. Double triggering was less likely to occur during day hours (RR: 0.88; 95% CI: 0.85–0.90; *p* < 0.001) and occurred most frequently in pressure control ventilation (PCV) mode (median: 3; IQR: 1–9 per hour). Ineffective effort was more likely to occur during day time (RR: 1.09; 95% CI: 1.05–1.13; *p* < 0.001), and occurred most frequently in PSV mode (median: 8; IQR: 2–29 per hour). The effect of sedatives and analgesics showed temporal patterns in DLNM. PVAs were not associated mortality and VAE in Cox regression models with time-varying covariates.

**Conclusions:** Our study showed that counts of PVAs were significantly influenced by time of the day, ventilation mode, ventilation settings (e.g., tidal volume and plateau pressure), and sedatives and analgesics. However, PVAs were not associated with the hazard of VAE or mortality after adjusting for protective ventilation strategies such as tidal volume, plateau pressure, and positive end expiratory pressure (PEEP).

## Introduction

Patient–ventilator asynchrony (PVA) is common in intensive care unit (ICU) patients (1, 2). PVA can be defined as a mismatch between patient respiratory effort and ventilator support. Most prevalent types of asynchrony include ineffective efforts, double triggering (DT), and early/late cycling off (3). Well-known risk factors for PVA include inappropriate level of inspiratory assist, ventilator mode, and the level of sedation (3). Several techniques have been used clinically to evaluate patient–ventilator interaction, including esophageal pressure, diaphragm electrical activity (4), and software algorithms analyzing ventilator flow and pressure curves (2). There is evidence showing that PVA is associated with adverse clinical outcomes, including mortality (5). However, previous epidemiological studies have important limitations. First, most techniques for the detection of PVA requires the physical presence of an expert physician at the bedside and is thus only feasible during short periods (3, 6–8). Second, risk factors were explored in a simplified time-fixed manner (9, 10). In reality, both well-known risk factors and PVAs are time varying; in addition, some risk factors may take time (lag) to take effect. In this situation, both the magnitude and time lag between exposure and PVA should be accounted for. Third, the association of PVA and mortality risk was mainly explored in small studies (5, 11), and the association was explored by dividing patients into groups with different degrees of PVA severity as represented by the asynchrony index (AI) (2). Since PVA is a time-varying covariate, it is important to appropriately account for the time-varying property of the PVA, while avoiding the immortal time bias (12).

The current study employed high-granularity data from multiparameter monitors and ventilators to explore the risk factors of PVA, the association with ventilator-associated events (VAEs), and mortality. We hypothesized that time of day, ventilation mode, ventilator settings, and sedatives could affect the PVA. In a multivariable regression model, we adjusted the sedatives and analgesics to see whether time of day was still independently associated with PVA. Secondly, we hypothesize that PVA has a negative impact on clinically important outcomes such as VAE and mortality.

## Methods

### Study Design and Setting

The study was conducted in an academic medical center from June 2019 to December 2019. The last follow-up date was on December 31, 2019, when the last patient was discharged home. Patients' electronic medical records (EMRs) were retrospectively reviewed. The study was approved by the ethics committee of the Sir Run Run Shaw Hospital (20190916-16). Informed consent was waived by the institutional review board due to the retrospective nature of the study. The study was conducted in accordance with the Helsinki declaration. The study was reported in accordance to the REporting of OBservational studies Conducted using Observational Routinely-collected Data (RECORD) checklist (13).

### Participants

Patients receiving invasive mechanical ventilation (IMV) at ICU admission were potentially eligible for the study. Patients were excluded if they (1) were younger than 15 years; (2) signed a do-not-resuscitate order; (3) were transferred from other ICUs for long-term care; (4) were terminally ill with an expected length of ICU stay of <48 h; (5) had no mechanical ventilation (MV) waveforms available. Since volume-controlled ventilation was seldom used in our institution (<5% ventilation hours), effective identification of PVA was impossible by our deep learning algorithms. Thus, patients with volume-controlled ventilation was excluded.

### Variables

Variables were extracted from EMR including demographics, reasons for MV, sequential organ failure assessment (SOFA) score, source of ICU admission, and vital status on hospital discharge. Time-varying covariates were recorded during MV, including VAE, ventilation mode, ventilator setting, sedatives, and analgesics. VAE was defined as either two or more baseline days of stable or decreasing daily minimum positive end expiratory pressure (PEEP) values followed by at least 2 days of daily minimum PEEP values 3 cm H_2_O above each of the two baseline days' values or two or more baseline days of stable or decreasing daily minimum FiO_2_ values followed by at least 2 days of daily minimum FiO_2_ values 0.20 above each of the 2 baseline days' values (14). VAE was used as a study end-point because (1) VAE can be included as a time-varying covariate in our longitudinal dataset; (2) it can be more objectively defined than ventilator-associated pneumonia; and (3) the impact of PVA on mortality might be mediated via VAE. Missing values were handled with single imputation.

### Identification of Four Types of Asynchrony

A one-dimensional interpretable convolutional neural network (1D-CNN) model was developed to detect DT, ineffective inspiratory effort during expiration (IEE), prolonged cycling (PC), and short cycling (SC). The model follows the classical AlexNet structure, which has excellent performance for image processing (15). The features in the ventilator waveforms were extracted by the convolutional layers, concatenated, and processed by a global averaging pooling (GAP) layer and a softmax layer for the final binary classification. The GAP layer allows us to highlight which segments contribute to the classification results mostly, thus providing a visual interpretation of the PVA classification. Individual deep learning models were developed under all ventilation modes. Under each ventilation mode, four models were established for detecting DT, IEE, PC, and SC. Each model uses the raw ventilator waveforms (airway pressure and flow) as input for a binary classification (PVA or non-PVA). Datasets were annotated by a group of clinical professionals for training and validating the models following the same approach proposed in our previous study (16). Fivefold cross-validation shows that the PVA recognition accuracy reached above 95% for all types of PVA in all the ventilation modes. Details of the data annotation, algorithm development, and validation are described in the ESM.

### Statistical Methods

Descriptive statistics were reported and compared by convention. Continuous data were expressed as mean and standard deviation (SD) or median and interquartile range (IQR) as appropriate. They were compared between survivors and non-survivors by using *t*-test or rank sum test. Categorical data were expressed as the number and percentage and were compared between different outcome groups by chi-square test or Fisher's exact test (17).

Potential risk factors associated with PVA such as ventilator mode, time of day, and ventilator settings were explored using the negative binomial regression because it is suitable for the description of the probabilities of the occurrence of whole numbers ≥0. Unlike Poisson regression, it does not require for the variance and the mean of the outcome count to be equivalent (18).

The association of sedatives/analgesics with PVA was explored using the distributed lag non-linear model (DLNM), which allows for lagged effect of these drugs (19). Drug exposure was considered in two dimensions of drug dose and time lag after the exposure. All other factors such as ventilator type, clock hours, and ventilator setting were adjusted in the model as a unidimensional variable.

The potential impact of PVA on clinical outcomes (VAE and mortality) was explored with the Cox regression model with time-varying covariates (20, 21). That is, the PVA counts were entered into the model for every hour before the occurrence of the outcome. Other time-varying covariates included ventilator parameters such as plateau pressure, PEEP, tidal volume, and work of breathing (WOB). Time-fixed variables included age, BMI, gender, admission type, reasons for MV, and SOFA score.

## Results

### Participants and Descriptive Data

A total of 160 patients were screened during the study period. After the exclusion of 14 patients due to missing waveform data, ventilation of <24 h, presence of volume-controlled ventilation, and presence of a do-not-resuscitate order, we finally included 146 patients for analysis. A total of 50,124 h involving 51,451,138 respiratory cycles was analyzed (e.g., an average of 51,451,138/50,124/60 = 17 cycles per minute). The overall mortality rate was 15.6%. Non-survivors showed greater SOFA [9.5 (7, 13) vs. 6.5 (5, 9); *p* = 0.009] and NUTRIC score (6.62 ± 2.2 vs. 4.94 ± 2.06; *p* = 0.077, Table 1), but there was no difference in mortality rate between VAE and non-VAE groups (Table 2). The VAE group showed longer ICU length of stay [21.82 (17.01, 29.82) vs. 12.24 (7.18, 18.99) days; *p* < 0.001] and MV duration [18.18 (13.83, 25.94) vs. 8.46 (5.93, 12.6) days; *p* < 0.001] than did the non-VAE group (Table 2).

**Table 1:** Comparisons between survivors and non-survivors.

**Variables**	**Total (*n* = 146)**	**Survivors (*n* = 123)**	**Non-survivors (*n* = 23)**	***p***
Age (years), median (IQR)	69 (56, 77)	67 (56.5, 75.5)	72 (54, 84.5)	0.289
BMI (kg/m^2^), median (IQR)	61.5 (33.25, 91.75)	64 (34.5, 93)	42 (31.5, 79)	0.267
Reasons for MV, *n* (%)				0.545
Cardiac disease	16 (11)	13 (11)	3 (13)	
Neuromuscular disease^#^	48 (33)	44 (36)	4 (17)	
Post-operation	17 (12)	13 (11)	4 (17)	
COPD	12 (8)	9 (7)	3 (13)	
Sepsis	30 (21)	25 (20)	5 (22)	
Systemic disease^*^	13 (9)	10 (8)	3 (13)	
Trauma	9 (6)	8 (7)	1 (4)	
SOFA, median (IQR)	7 (5, 10)	6.5 (5, 9)	9.5 (7, 13.25)	0.009
APACHE II, mean ± SD	22.42 ± 8.34	22.06 ± 8	24.22 ± 9.91	0.334
VAE, *n* (%)	26 (18)	19 (15)	7 (30)	0.132
ICU LOS (days), median (IQR)	12.91 (7.72, 22.12)	12.91 (7.95, 22.66)	12.48 (5.92, 19.38)	0.271
NUTRIC score, mean ± SD	5.27 ± 2.17	4.94 ± 2.06	6.62 ± 2.2	0.077

*MV, mechanical ventilation; IQR, interquartile range; SD, standard deviation; SOFA, sequential organ failure assessment; COPD, chronic obstructive pulmonary disease; APACHE, Acute Physiology and Chronic Health Evaluation; LOS, length of stay; ICU, intensive care unit; NUTRIC, nutrition Risk in the Critically ill*.

# *Neuromuscular disease included disorders such as respiratory failure caused by neuromuscular disorder like stroke and Guillain–Barre syndrome*.

* *Systemic disease included autoimmune diseases such as SLE*.

**Table 2:** Clinical outcomes between VAE and non-VAE groups.

**Variables**	**Total (*n* = 147)**	**Non-VAE (*n* = 121)**	**VAE (*n* = 26)**	***p***
ICU LOS (days), median (IQR)	12.91 (7.72, 22.12)	12.24 (7.18, 18.99)	21.82 (17.01, 29.82)	<0.001
MV days, median (IQR)	9.93 (6.05, 15.9)	8.46 (5.93, 12.6)	18.18 (13.83, 25.94)	<0.001
Mortality, *n* (%)	23 (16)	16 (13)	7 (27)	0.132

*IQR, interquartile range; MV, mechanical ventilation; LOS, length of stay; ICU, intensive care unit; VAE, ventilator associated events*.

### The Performance of the PVA Detection Models Under Different Ventilation Modes

**Figure 1:**
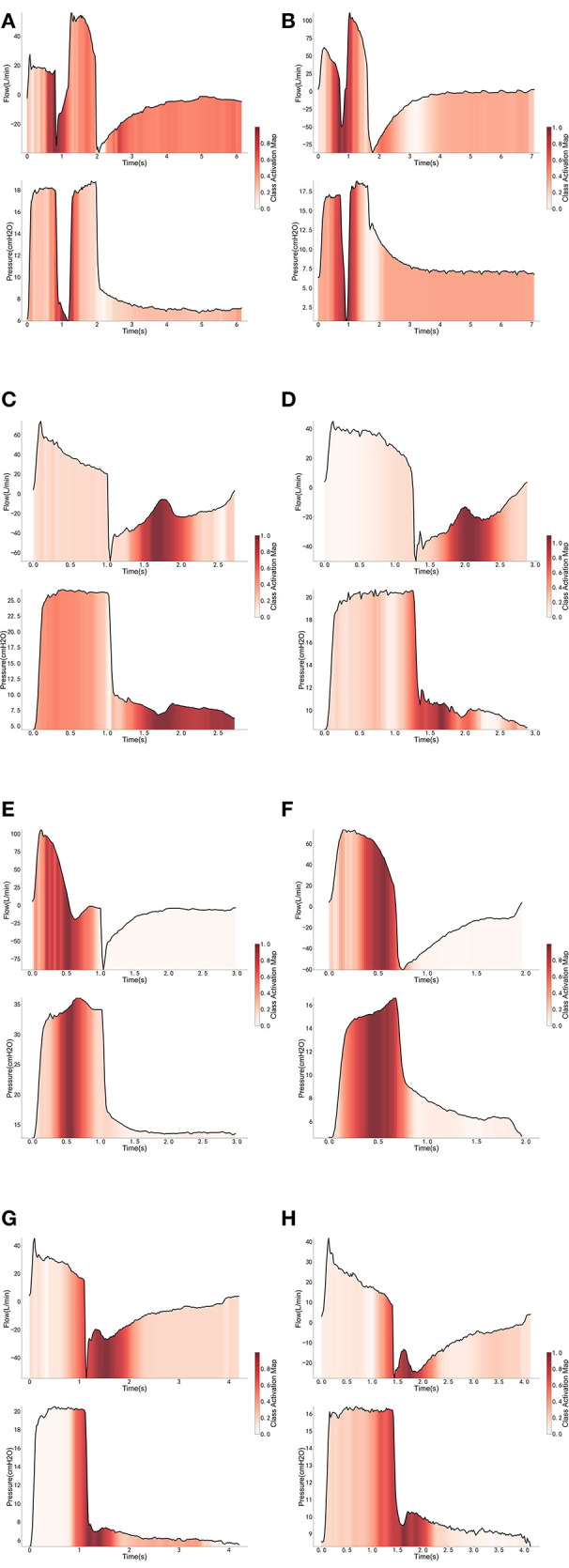
Interpretation of the cycles classified as PVA under PCV mode **(A,C,E,G)** and PSV mode **(B,D,F,H)**.

Eight independent binary classifiers were developed for different types of PVA under different ventilation modes, i.e., H_PCV−IEE_, H_PCV−DT_, H_PCV−Prol_, H_PCV−Short_, H_PSV−IEE_, H_PSV−DT_, H_PSV−Prol_, and H_PSV−Short_. The performance of the models was evaluated by a fivefold cross-validation. The average accuracy, sensitivity, and specificity are given in Table 3. We intended to interpret the PVA recognition using a class activation map (CAM) technique (22). The technique replaced the FC layer in the CNN model with a GAP layer to allow visualization of the sections that the CNN model focuses on. In other words, the sections that contribute mostly to the classification results will be highlighted. In this way, we may understand why the CNN model decides a certain breath manifests PVA. The interpretation of the classification under the three involved ventilation modes is illustrated in Figure 1.

**Table 3:** The performance of the PVA detection models under different ventilation modes.

	**Modes**	**ACC**	**SEN**	**SPE**
IEE	PCV	0.972 ± 0.001	0.975 ± 0.003	0.969 ± 0.003
	PSV	0.993 ± 0.003	0.994 ± 0.002	0.991 ± 0.005
DT	PCV	0.986 ± 0.001	0.992 ± 0.004	0.979 ± 0.006
	PSV	0.985 ± 0.002	0.986 ± 0.008	0.984 ± 0.006
Prolonged cycling	PCV	0.979 ± 0.002	0.977 ± 0.007	0.982 ± 0.005
	PSV	0.973 ± 0.004	0.973 ± 0.004	0.973 ± 0.008
Short cycling	PCV	0.970 ± 0.005	0.975 ± 0.008	0.966 ± 0.004
	PSV	0.985 ± 0.003	0.987 ± 0.003	0.984 ± 0.005

*ACC, accuracy; SPE, specificity; SEN, sensitivity; PCV, pressure control ventilation; PSV, pressure support ventilation; DT, double triggering; IEE, ineffective effort*.

### Risk Factors of PVA

**Figure 2:**
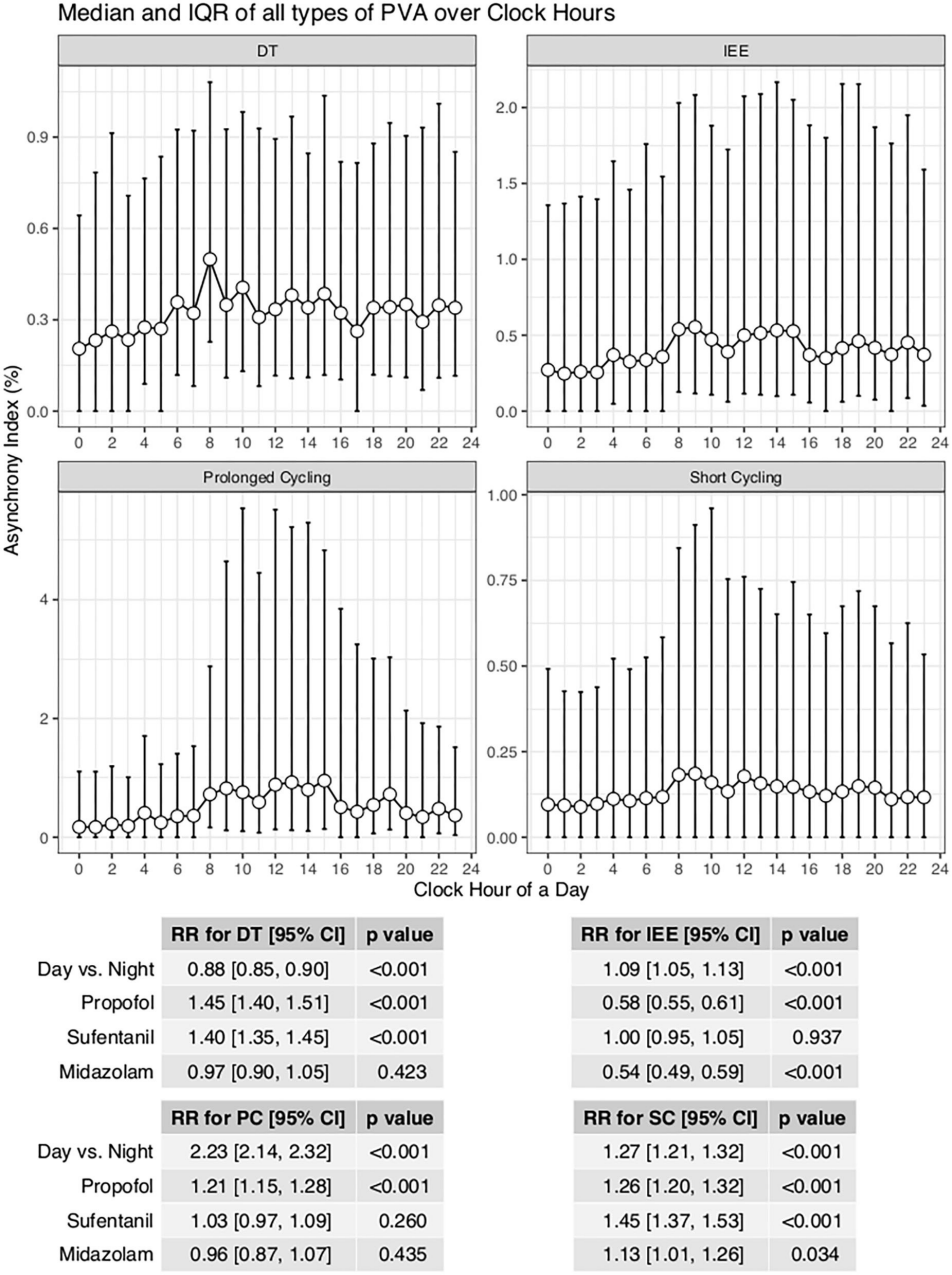
Impact of day hours on four types of asynchrony. AI was defined as the percentage of respiratory cycles with the presence of relevant types of PVA. A negative binomial regression model was built to adjust for the confounding effect of analgesics and sedatives. IEE, ineffective effort; DT, double triggering; SC, short cycling, PC, prolonged cycling.

**Figure 3:**
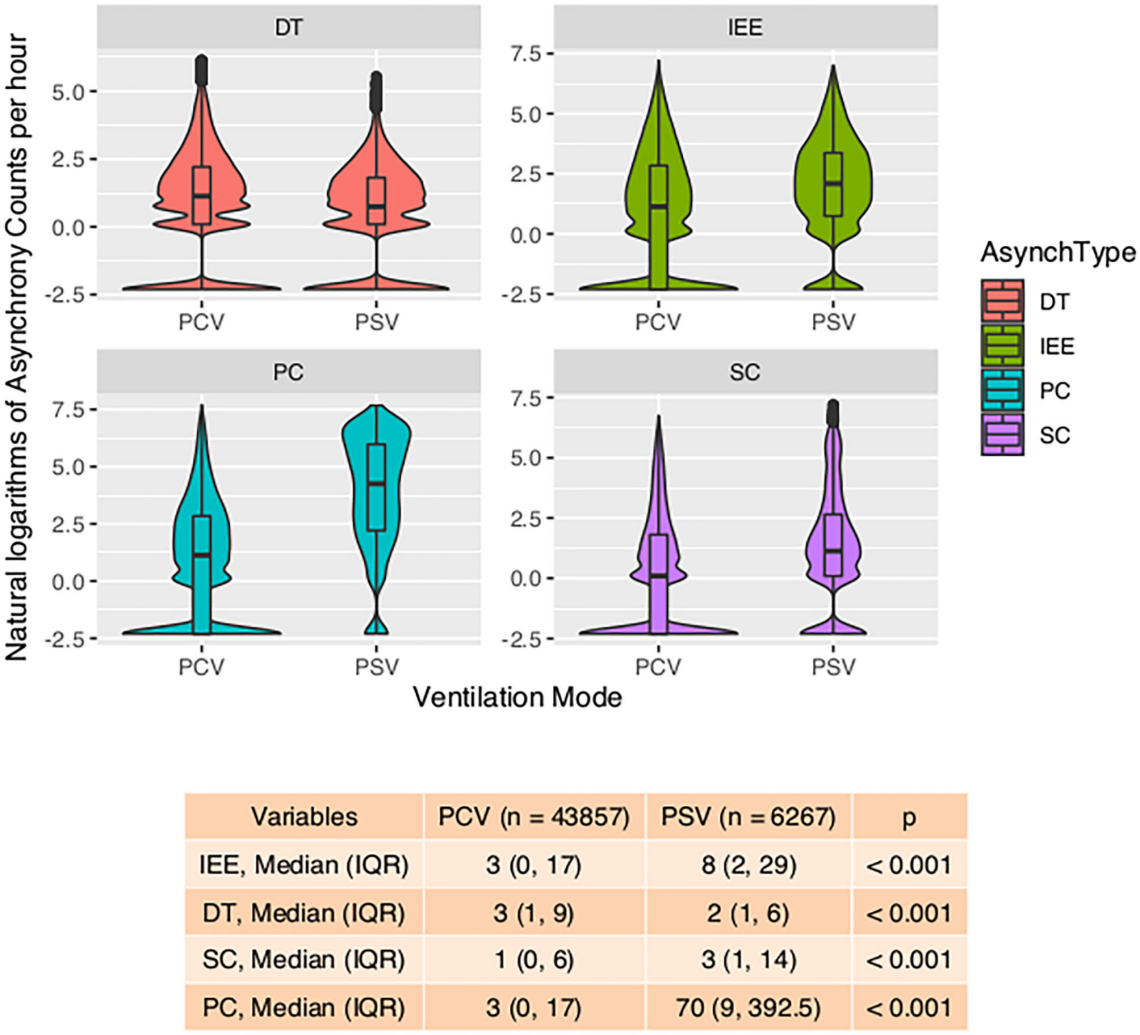
Violin plot showing the impact of ventilation mode on four types of asynchrony. Violin-and-box plots are used to visualize the distribution of the asynchrony counts (transformed by natural logarithms) and their probability density. The table at the bottom shows the number of asynchrony counts per hour. IEE, ineffective effort; DT, double triggering; SC, short cycling; PC, prolonged cycling; PCV, pressure control ventilation; PSV, pressure support ventilation.

**Figure 4:**
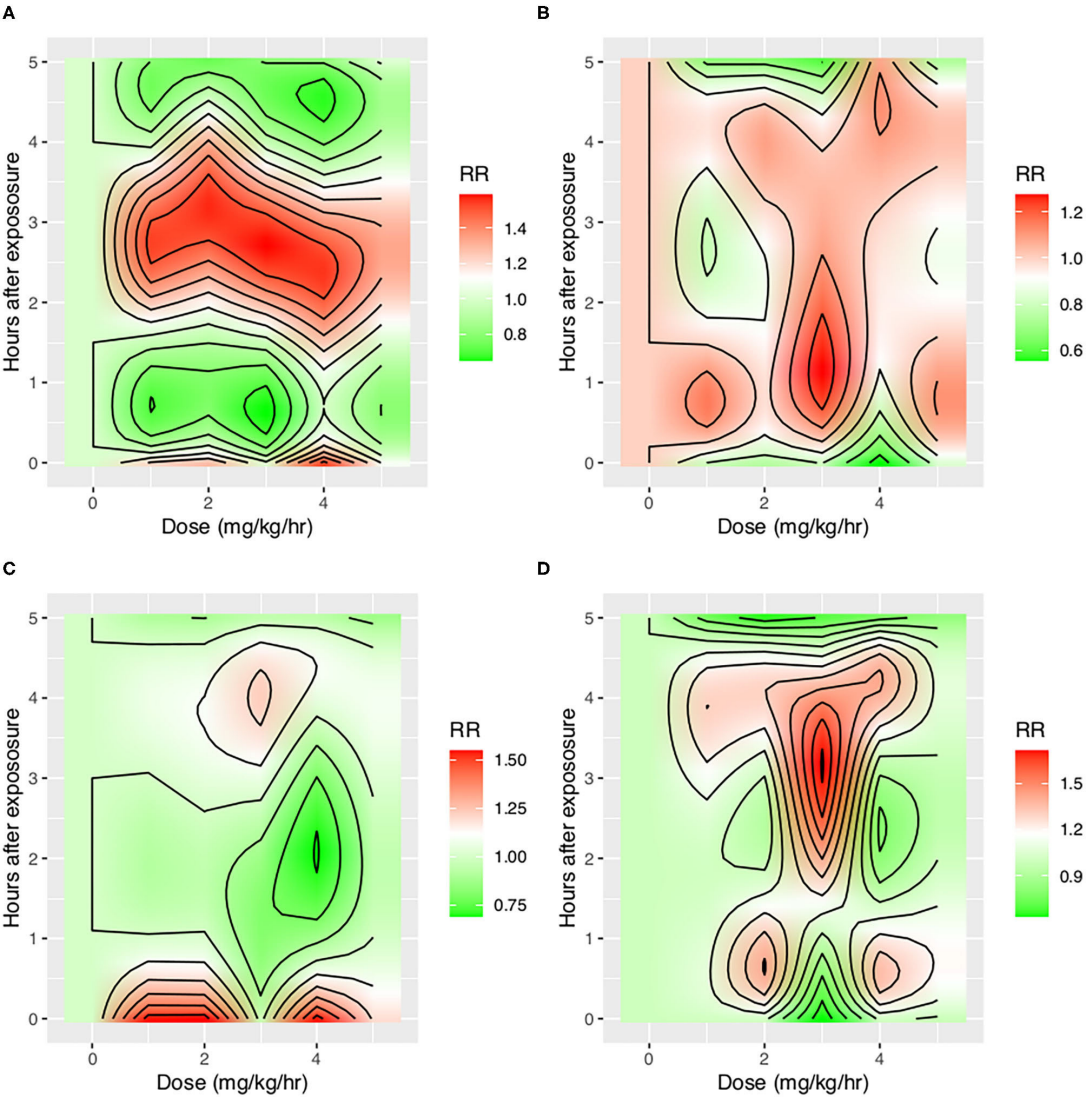
Impact of propofol on four types of asynchrony. Propofol was entered into the distributed lag non-linear model with two dimensions: dose and time lag. The y-axis shows the time after instantaneous exposure of propofol, so the drug was assumed to be discontinued after a certain dose exposure. Other covariates including tidal volume, WOB, PEEP, plateau pressure, mode of ventilation, and day hours were adjusted. The red color shows increased risk of asynchrony, and the green color shows reduced risk of asynchrony. **(A)** Impact on DT, **(B)** Impact on IEE, **(C)** Impact on PC, and **(D)** Impact on SC. IEE, ineffective effort; DT, double triggering; SC, short cycling; PC, prolonged cycling.

With the ML model used to detect PVA, the occurrence of PVA varied depending on the time of day (Figure 2). DT, PC, and SC were less likely to occur during 0–3 o'clock (Figure 2). To examine whether the difference in the effect of day vs. night was attributable to the difference of the use of sedatives and analgesics, we adjusted for the use of analgesics and sedatives in the negative binomial regression model (Figure 2). DT was less likely to occur during day hours (RR: 0.88; 95% CI: 0.85–0.90; *p* < 0.001). IEE (RR: 1.09; 95% CI: 1.05–1.13; *p* < 0.001), PC (RR: 2.23; 95% CI: 2.14–2.32; *p* < 0.001), and SC (RR: 1.27; 95% CI: 1.21–1.32; *p* < 0.001) were more likely to occur during daytime. Ventilator mode (PSV vs. PCV) was also significantly associated with the incidence of PVA (Figure 3). DT was more likely to occur in PCV than in PSV (median [IQR]: 3 [1–9] vs. 2 [1–6] per hour), whereas IEE occurred more frequently in PSV than in PCV (8 [2–29] vs. 3 [0–17] per hour). In the DLNM model, each drug was considered in two dimensions of dosage and time after exposure (time lag after instantaneous exposure to a certain dose of the drug). Propofol was able to reduce the incidence of DT 30–60 min after exposure (i.e., the drug was discontinued after infusion at a dose of 1–3 mg/kg/h); however, the count of DT increased after 2–4 h following discontinuation after infusion at a dose of 1–4 mg/kg/h (Figure 4). The effects of midazolam and sufentanil are shown in SEM (Supplementary Figures 1, 2). Finally, all risk factors were entered into negative binomial regression models with each asynchrony type as the response variable (Table 4). The result showed that day hour, ventilator mode, tidal volume, PEEP, and WOB were all associated with PVAs.

**Table 4:** Negative binomial regression model exploring the risk factors for the four types of asynchronies.

**Variables**	**RR for IEE (95% CI)**	***p***	**RR for DT (95% CI)**	***p***	**RR for SC (95% CI)**	***p***	**RR for PC (95% CI)**	***p***
Day hours (night as reference)^*^	1.063 (1.026, 1.101)	<0.001	0.994 (0.964, 1.024)	0.666	0.963 (0.923, 1.006)	0.084	1.243 (1.196, 1.293)	<0.001
Ventilation mode (PCV as reference)	1.186 (1.111, 1.267)	<0.001	0.402 (0.381, 0.424)	<0.001	1.388 (1.285, 1.499)	<0.001	4.398 (4.103, 4.715)	<0.001
**TV (<6 ml/kg as reference)**								
6–8 ml/kg	0.654 (0.612, 0.699)	<0.001	0.718 (0.684, 0.753)	<0.001	1.139 (1.06, 1.223)	<0.001	1.43 (1.339, 1.526)	<0.001
8–10 ml/kg	0.425 (0.392, 0.46)	<0.001	0.541 (0.511, 0.573)	<0.001	1.47 (1.349, 1.6)	<0.001	1.87 (1.725, 2.026)	<0.001
>10 ml/kg	0.239 (0.215, 0.267)	<0.001	0.419 (0.387, 0.454)	<0.001	1.519 (1.346, 1.715)	<0.001	5.159 (4.575, 5.818)	<0.001
**WOB (<10 J/ml/kg as reference)**								
10–15 J/ml/kg	1.26 (1.182, 1.343)	<0.001	0.948 (0.904, 0.995)	0.04	0.473 (0.442, 0.506)	<0.001	0.415 (0.388, 0.444)	<0.001
15–20 J/ml/kg	1.167 (1.068, 1.275)	<0.001	1.239 (1.162, 1.322)	<0.001	0.556 (0.507, 0.61)	<0.001	0.216 (0.197, 0.237)	<0.001
>20 J/ml/kg	0.867 (0.776, 0.969)	0.008	2.114 (1.949, 2.292)	<0.001	1.206 (1.067, 1.364)	0.003	0.154 (0.136, 0.174)	<0.001
**PEEP (≤5 cm H**_**2**_**O as reference)**								
5–10 cm H_2_O	0.638 (0.61, 0.668)	<0.001	1.236 (1.191, 1.282)	<0.001	1.218 (1.155, 1.286)	<0.001	1.443 (1.369, 1.521)	<0.001
>10 cm H_2_O	1.063 (0.94, 1.205)	0.313	2.018 (1.823, 2.238)	<0.001	6.702 (5.722, 7.869)	<0.001	4.446 (3.875, 5.116)	<0.001
**Plateau pressure (<20 cm H**_**2**_**O as reference)**								
20–30 cm H_2_O	1.39 (1.317, 1.467)	<0.001	0.64 (0.613, 0.668)	<0.001	0.538 (0.506, 0.571)	<0.001	1.354 (1.271, 1.441)	<0.001
>30 cm H_2_O	0.768 (0.703, 0.838)	<0.001	0.318 (0.296, 0.341)	<0.001	0.079 (0.071, 0.088)	<0.001	0.96 (0.87, 1.06)	0.401

*RR, relative risk; CI, confidence interval; DT, double triggering; IEE, ineffective effort; SC, short cycling; PC, prolonged cycling; PEEP, positive end expiratory pressure; WOB, work of breathing; VCV, volume control ventilation; PCV, pressure control ventilation; VC+, volume control plus; APRV, airway pressure release ventilation; PSV, pressure support ventilation; CPAP, continuous positive airway pressure; TV, tidal volume. Four negative binomial regression models were built by using each type of asynchrony as the dependent variable. All variables in the table were entered into the models to adjust for confounding effects*.

* *Day hours were categorized by visually inspecting the asynchrony–day hour trend curve*.

### Impact of PVA on Clinical Outcomes

PVA was entered into a Cox regression model as a time-varying covariate. After adjusting for baseline characteristics and other time-varying covariates, PVA was not associated with increased risk of mortality or VAE (Table 5). Interestingly, high plateau pressure (>30 cm H_2_O) was a significant risk factor for both mortality (HR: 26.95; 95% CI: 1.95–372.59; *p* = 0.014) and VAE (HR: 9.30; 95% CI: 1.34–64.38; *p* = 0.024). Large tidal volume (>10 ml/kg) was associated with increased risk of fatality (HR: 11.22; 95% CI: 1.27–99.28; *p* = 0.03). Other significant risk/protective factors for VAE were admission from emergency department (HR: 0.23; 95% CI: 0.07–0.83; *p* = 0.024), SOFA (HR: 1.21; 95% CI: 1.03–1.43; *p* = 0.019), and MV due to systematic disorders such as systematic lupus erythematosus (HR: 0.04; 95% CI: 0.00–0.44; *p* = 0.008).

**Table 5:** Cox regression model with time-varying covariates.

**Variables**	**HR for VAE (95% CI)**	***p***	**HR for mortality (95% CI)**	***p***
Age (for every 1-year increase)	0.97 (0.92, 1.02)	0.246	1.01 (0.97, 1.05)	0.509
BMI (for every 1-point increase)	0.98 (0.96, 1.00)	0.010	1.00 (0.99, 1.01)	0.674
Gender (female as reference)	0.83 (0.25, 2.74)	0.765	1.96 (0.46, 8.38)	0.364
**Admission type (from ward as reference)**				
Emergency room	0.23 (0.07, 0.83)	0.024	2.41 (1.05, 5.52)	0.038
Others	0.84 (0.13, 5.49)	0.859	1.67 (0.30, 9.20)	0.558
SOFA (for every 1-point increase)	1.21 (1.03, 1.43)	0.019	1.23 (0.99, 1.53)	0.065
**Reasons for MV (cardiac disease as reference)**				
Neuromuscular disease	0.35 (0.04, 3.13)	0.347	0.64 (0.10, 4.18)	0.644
Post-operation	0.25 (0.04, 1.75)	0.162	0.78 (0.11, 5.41)	0.804
COPD	0.39 (0.06, 2.64)	0.331	1.87 (0.34, 10.42)	0.474
Sepsis	0.31 (0.07, 1.33)	0.114	1.75 (0.35, 8.63)	0.494
Systemic disease	0.04 (0.00, 0.44)	0.008	1.19 (0.26, 5.46)	0.821
Trauma	2.98 (0.38, 23.19)	0.297	1.24 (0.12, 12.95)	0.857
IEE (for every increase per hour)	1.00 (1.00, 1.01)	0.250	1.00 (0.99, 1.00)	0.139
DT (for every increase per hour)	1.00 (0.99, 1.01)	0.687	1.00 (0.98, 1.01)	0.677
SC (for every increase per hour)	1.02 (0.99, 1.04)	0.183	0.83 (0.61, 1.13)	0.239
PC (for every increase per hour)	0.69 (0.40, 1.21)	0.197	0.70 (0.39, 1.27)	0.242
**WOB (<10 J/ml/kg as reference)**				
10–15 J/ml/kg	0.27 (0.03, 2.12)	0.215	0.27 (0.02, 3.04)	0.289
15–20 J/ml/kg	0.88 (0.12, 6.54)	0.899	0.14 (0.01, 2.43)	0.179
>20 J/ml/kg	0.98 (0.09, 10.63)	0.988	0.06 (0.00, 1.91)	0.111
**TV (<6 ml/kg as reference)**				
6–8 ml/kg	2.43 (0.13, 46.21)	0.554	3.07 (0.77, 12.30)	0.113
8–10 ml/kg	2.86 (0.14, 60.39)	0.499	2.26 (0.38, 13.34)	0.370
>10 ml/kg	5.31 (0.19, 145.37)	0.323	11.22 (1.27, 99.28)	0.030
**Plateau pressure (<20 cm H**_**2**_**O as reference)**				
20–30 cm H_2_O	4.03 (1.20, 13.58)	0.025	5.63 (0.64, 49.22)	0.118
>30 cm H_2_O	9.30 (1.34, 64.38)	0.024	26.95 (1.95, 372.59)	0.014

*HR, hazard ratio; CI, confidence interval; WOB, work of breathing; TV, tidal volume; IEE, ineffective effort; DT, double triggering; SC, short cycling; PC, prolonged cycling; SOFA, sequential organ failure assessment; COPD, chronic obstructive pulmonary disease; BMI, body mass index*.

## Discussion

This is the most comprehensive study to investigate the epidemiology and clinical consequences of PVA in ICU patients. The main findings can be summarized as follows: First, our study shows that day hours, ventilation mode, ventilator parameters, sedatives, and analgesics were important risk factors for all types of asynchrony. The effect of sedatives and analgesics showed time-dependent patterns. Second, PVAs were not associated with either VAE or mortality after adjusting for covariates. Third, ventilator parameters such as tidal volume and plateau pressure were significantly associated with VAE and mortality in a Cox regression model with time-varying covariates. Our study indicates that although protective ventilation strategies such as low tidal volume and low plateau pressure were associated with increased PVA, it is unwise to increase the TV and plateau pressure in order to reduce PVA, because increasing TV and plateau pressure would increase the hazard of VAE and mortality. Our deep learning algorithm can be used in a standard ICU for real-time monitoring of PVAs. High frequency or intensity of PVAs can trigger warnings from the machine, and measures can be taken to modify some risk factors as identified in our study.

One strength of our study was that different types of PVAs were identified by using deep learning algorithms and were analyzed separately (16). We believe that different PVAs have different underlying mechanisms, and risk factors and its consequences can be different (3). Previous studies have analyzed PVAs as a composite outcome that all types of PVAs were aggregated as a single index called AI (2, 10). Our study found that risk factors for different PVAs were different. For example, while IEE, PC, and SC were more likely to occur during daytime, DT was less likely to occur during daytime after adjustment for the use of sedatives and analgesics (Figure 1). Pathophysiologically, DT is the result of high inspiratory demand and excessive inspiratory effort (23). Inspiratory demand can be high during daytime because of the diurnal variation pattern (24). Furthermore, patients are more likely to be awake and influenced by medical procedures during day hours. Propofol also showed differing effects on IEE and DT. At 30–60 min after propofol discontinuation, the risk of DT decreased, but the risk of IEE increased (Figure 3). Propofol could reduce patient inspiratory efforts and thus DT. Recall that DT could be the result of excessive inspiratory efforts (25). However, when there is too much sedative, some normal inspiratory efforts are reduced such that they fail to trigger a respiratory cycle, leading to increased IEE. Such differing effects on different types of PVAs were also noted in another randomized controlled trial (26).

A novel finding in our study was that the effect of sedatives and analgesics on PVA followed distinct temporal patterns. Although previous studies have shown that sedatives were associated with reduced IEE (9), data from 1 day were binned in their studies, making it difficult to explore the causal/temporal relationship of sedatives and PVA. For example, the attending physician may give more sedative for a patient with increased PVAs, and sedatives may also change the risk of PVAs. The sedatives and PVAs construct a cyclic causal diagram. Our study employed DLNM to explore the temporal effect of sedatives on different types of PVA. It was interesting to find that the risk of DT first decreased at 30–60 min after propofol infusion and then increased at 3–4 h after propofol discontinuation, which was probably due to the short half-life of the drug (30–60 min) and increased risk of delirium after propofol infusion (27). In a controlled experimental study, Vaschetto and colleagues showed that deep propofol sedation increased asynchronies, while light sedation did not (25). Our finding was consistent with Vaschetto's study in that high-dose propofol was associated with increased risk of DT at the same hour of propofol infusion (Figure 3).

Our study was the first to systematically explore the association of protective ventilation strategy on PVAs. We found that protective ventilation strategies such as low tidal volume, low plateau pressure, and high PEEP were all significantly associated with the risk of PVAs, after adjusting for other risk factors in negative binomial regression models. Other studies also observed some patients with strong inspiratory effort and patient–ventilator mismatch when the tidal volume was given below 6.5 ml/kg (28). The protective ventilation strategy usually cannot meet patient requirements, and thus PVAs are common; thus, more sedatives and neuromuscular blocking agents are usually required to deliver protective ventilation strategies (29). In Cox regression models with PVAs and ventilation parameters as time-varying covariates, we did not find independent associations between PVAs and the hazard of mortality and VAE, which was consistent with other studies (10, 11, 30). However, this finding does not mean that we shall no longer pay attention to the PVA phenomenon. The reasons for our study not finding statistically significant results might be that there are numerous factors that can influence mortality and that the effect size of a single variable is very small. The sample size or statistical power must be very large to reach the statistical significance level. PVA can cause patient discomfort and may be a sign of inappropriate ventilation setting. However, the use of protective ventilation strategy was associated with mortality and VAE. These results indicate that we should not increase tidal volume or plateau pressure in order to reduce PVAs. If VAE is the primary concern, we could use sedatives and neuromuscular blocking agents to safely deliver the protective ventilation strategy while avoiding PVAs (31).

Several limitations must be acknowledged in the study. First, reverse triggering was not distinguished from DT, because we did not have data on esophageal pressure monitoring. There has been evidence that reverse triggering is different from other types of PVAs from a pathophysiological view (3, 32). Ideally, it should be analyzed independently. Clinical findings of the present study are based on the accuracy of the method for detecting PVA coming from a machine learning model, and the results are limited by its accuracy. Second, the study included heterogeneous MV patients including those with ARDS and COPD. Although we have adjusted our results by disease type, the sample sizes in some disease groups were limited. Third, the study was carried out in a single center, and it is unknown whether the results are generalizable to other hospitals. The limited sample size and small number of mortality event make our model preliminary, especially the results related to the mortality outcome. The model should be verified in studies with a larger sample size. Finally, the models trained in our study were not externally validated. Thus, further studies are required to validate current findings.

In conclusion, with the ML model used to detect PVA, our study showed that counts of PVAs were significantly influenced by day hours, ventilation mode, ventilation parameters, and the use of sedatives and analgesics. However, PVAs were not associated with the hazard of VAE and mortality after adjusting for protective ventilation strategies such as tidal volume, plateau pressure, and PEEP.

## Take Home Message

- Our study showed that counts of PVAs were significantly influenced by time of day, ventilation mode, ventilation settings (e.g., tidal volume and plateau pressure), and sedatives and analgesics.
- PVAs were not associated with the hazard of VAE or mortality after adjusting for protective ventilation strategies such as tidal volume, plateau pressure, and PEEP.

## Data Availability Statement

The datasets used and/or analyzed during the current study are available from the corresponding author on reasonable request.

## Ethics Statement

The study was approved by the ethics committee of Sir Run Run Shaw hospital (20190916-16). Written informed consent for participation was not required for this study in accordance with the national legislation and the institutional requirements.

## Author Contributions

ZZ and HG conceived the idea, performed the analysis, and drafted the manuscript. KD, JW, and LJ collected the data. LZ, YZ, LF, and QP analyzed respiratory mechanics using deep learning methods. LH interpreted the results and helped revise the manuscript. All authors contributed to the article and approved the submitted version.

## Conflict of Interest

The authors declare that the research was conducted in the absence of any commercial or financial relationships that could be construed as a potential conflict of interest.
