# Supplementary material for: Risk Factors for Patient–Ventilator Asynchrony and Its Impact on Clinical Outcomes: Analytics Based on Deep Learning Algorithm
Source: Front Med (Lausanne). 2020 Nov 25;7:597406. doi: 10.3389/fmed.2020.597406 (PMC7724969; doi:10.3389/fmed.2020.597406)
Supplement: Supplementary file 1 [file Data_Sheet_1.PDF]

Electronic Supplemental Material for Epidemiology of patient-ventilator asynchrony and its impact on clinical outcomes

Table of Contents

Automatic detection of patient-ventilator asynchrony **.......... 2**

Data Collection 2

Data annotation 2

Model development 3

Table S1. Comparisons between survivors and non-survivors... *Error! Bookmark not defined.*

Table S2. Clinical outcomes between VAE and non-VAE groups *Error! Bookmark not defined.*

Figure S1. Impact of Midazolam on four types of asynchrony. 7

Figure S2. Impact of Sufentanil on four types of asynchrony. 8

Automatic detection of patient-ventilator asynchrony

In this study, we detected four types of patient-ventilator asynchrony. Double-triggering (DT) happens when the inspiratory effort sustains beyond the ventilator inspiratory time and triggers a second inspiration [1]. It is characterized by a very short expiration between two consecutive inspirations. Ineffective inspiratory efforts during expiration (IEE) indicates the inspiratory efforts that fail to trigger a breath in the expiratory phase. It manifests a reversal of flow accompanied with reduced airway pressure in the expiration [2]. Short cycling and prolonged cycling indicate that the inspiration time setting of ventilators is either shorter or longer than the patient inspiration time. Short cycling exhibits an early reversal of flow together with pressure decrease. Prolonged cycling shows a sharp pressure increase in the end of inspiration [3]. We detected these four types of asynchronies using an interpretable deep learning approach.

Data Collection

The ventilator waveforms were collected from 20 invasive ventilated adults in the ICUs of Sir Run Run Shaw Hospital of Zhejiang University of January 2020 using a ventilator information system (RespCare^{TM}, ZhiRuiSi, Hangzhou, China). Only the patients ventilated using PB840 (Covidien, U.S.) were included. Two types of ventilation modes, i.e. pressure control ventilation (PCV) and pressure support ventilation (PSV) were considered. Volume control ventilation was not considered because PCV was much more widely used in the investigated center than VCV.

Data annotation

The waveforms were annotated by clinical professionals following the protocol used in our previous study [5]. In brief, a group of junior professionals (5 respiratory therapists with 3-5 years clinical experience) annotated the waveforms. A group of senior professionals (respiratory therapists with clinical experience above 10 years) reviewed the annotations and made the final decision. Each breath was labeled as “DT”, “IEE”, “Short cycling”, “Prolonged cycling” or “Others”, which means the breath does not belong to any considered types of asynchrony. For DT cycles, we gave “DT” labels to two consecutive cycles. In order to establish binary classifiers for each type of asynchrony, we prepared PVA and non-PVA cycles as positive and negative samples respectively for each classifier. Taking the classifier for IEE detection as an example, we took IEE cycles as the positive samples and all the non-IEE cycles (including DT, short and prolonged cycling, and others) as negative samples. The dataset was balanced by randomly removing samples from the major group. The statistics of the annotated ventilator waveforms are given in Table e1.

Table e1 Information of the dataset

Ventilation Mode	Asynchrony Type	Amount of Data (Async/Non-Async)
**PCV**	IEE	7696/8254
	DT	4639/4693
	Prolonged cycling	7521/7591
	Short cycling	4407/4941
**PSV**	IEE	7996/7989
	DT	2204/2279
	Prolonged cycling	6317/6155
	Short cycling	3247/3373

Model development

We proposed an interpretable deep learning method to detect DT and IEE. A one-dimensional convolutional neural network (1D-CNN) was developed. The CNN architecture follows the AlexNet [6], which is a widely used deep learning architecture for image processing. A gradient class activation mapping (CAM) approach was developed to interpret the results by highlighting the segments that contribute mostly to the classification. The architecture of the deep learning model is given in Figure e1. The detailed network configurations we used on the proposed 1D-CNN are shown in Table e2. Airway pressure and flow time series were separately fed to two parallel 1D-AlexNets to extract features from the raw signals. The extracted features were concatenated and processed by a global averaging pooling (GAP) layer and a softmax layer for the final binary classification. The weights in the GAP layer can be used to produce a time-dependent CAM to indicate which segments contribute to the classification results mostly. The deep learning model was developed in Python using Keras as the backend deep learning library. The specifications of the workstation used for training the models consisted of Intel Core i7-8700 CPU, 16 GB of memory, and an NVIDIA GeForce GTX 1060 GPU.

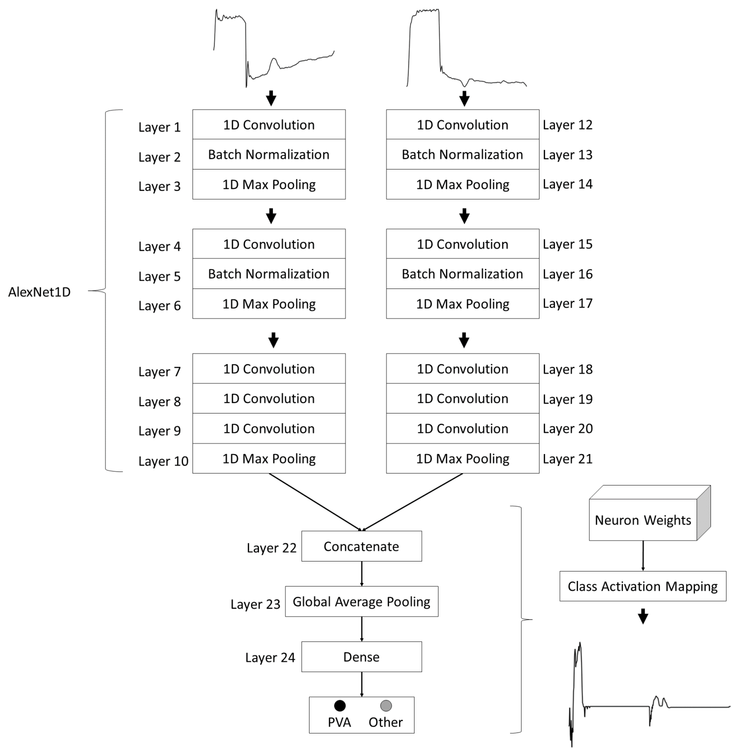

Figure e1 Architecture of the interpretable deep learning model

Table e2 Layer details and parameters used for the proposed 1D-CNN model

Layers	Types	Activation function	Output shapes	Size of kernel	No. of filters	Stride	No. of parameters
0 & 11	Input	-	300 x 1	-	-	-	0
1 & 12	1D Convolution	Relu	75 x 96	11 x 1	96	4	1152
2 & 13	BatchNormalization	-	75 x 96	-	-	-	384
3 & 14	1D Max Pooling	-	38 x 96	3 x 1	-	2	0
4 & 15	1D Convolution	Relu	38 x 256	5 x 1	256	1	123136
5 & 16	BatchNormalization	-	38 x 256	-	-	-	1024
6 & 17	1D Max Pooling		19 x 256	3 x 1		2	
7 & 18	1D Convolution	Relu	19 x 384	3 x 1	384	1	295296
8 & 19	1D Convolution	Relu	19 x 384	3 x 1	384	1	442752
9 & 20	1D Convolution	Relu	19 x 256	3 x 1	256	1	295168
10 & 21	1D Max Pooling	-	10 x 256	3 x 1	-	2	0
22	Concatenate	-	20 x 256	-	-	-	0
23	Global Average Pooling	-	256	-	-	-	0
24	Dense	Softmax	2	-	-	-	512
						Total	2318336

References

[1] C. Subira, C. de Haro, R. Magrans, R. Fernandez, L. Blanch, Minimizing Asynchronies in Mechanical Ventilation: Current and Future Trends, Respir. Care, 63 (2018) 464-478.
[2] L. Blanch, B. Sales, J. Montanya, U. Lucangelo, O. Garcia-Esquirol, A. Villagra, E. Chacon, A. Estruga, M. Borelli, M.J. Burgueño, J.C. Oliva, R. Fernandez, J. Villar, R. Kacmarek, G. Murias, Validation of the Better Care® system to detect ineffective efforts during expiration in mechanically ventilated patients: a pilot study, Intensive Care Med., 38 (2012) 772-780.
[3] B. Gholami, T.S. Phan, W.M. Haddad, A. Cason, J. Mullis, L. Price, J.M. Bailey, Replicating human expertise of mechanical ventilation waveform analysis in detecting patient-ventilator cycling asynchrony using machine learning, Comput. Biol. Med., 97 (2018) 137-144.
[4] A.W. Thille, P. Rodriguez, B. Cabello, F. Lellouche, L. Brochard, Patient-ventilator asynchrony during assisted mechanical ventilation, Intensive Care Med., 32 (2006) 1515-1522.
[5] L. Zhang, K. Mao, K. Duan, S. Fang, Y. Lu, Q. Gong, F. Lu, Y. Jiang, L. Jiang, W. Fang, X. Zhou, J. Wang, L. Fang, H. Ge, Q. Pan, Detection of patient-ventilator asynchrony from mechanical ventilation waveforms using a two-layer long short-term memory neural network, Comput. Biol. Med., 120 (2020) 103721.
[6] A. Krizhevsky, I. Sutskever, G.E. Hinton, ImageNet Classification with Deep Convolutional Neural Networks, neural information processing systems, 2012, pp. 1097-1105.
[7] L. Blanch, A. Villagra, B. Sales, J. Montanya, U. Lucangelo, M. Luján, O. García-Esquirol, E. Chacón, A. Estruga, J.C. Oliva, A. Hernández-Abadia, G.M. Albaiceta, E. Fernández-Mondejar, R. Fernández, J. Lopez-Aguilar, J. Villar, G. Murias, R.M. Kacmarek, Asynchronies during mechanical ventilation are associated with mortality, Intensive Care Med., 41 (2015) 633-641.

Figure S1. Impact of Midazolam on four types of asynchrony.

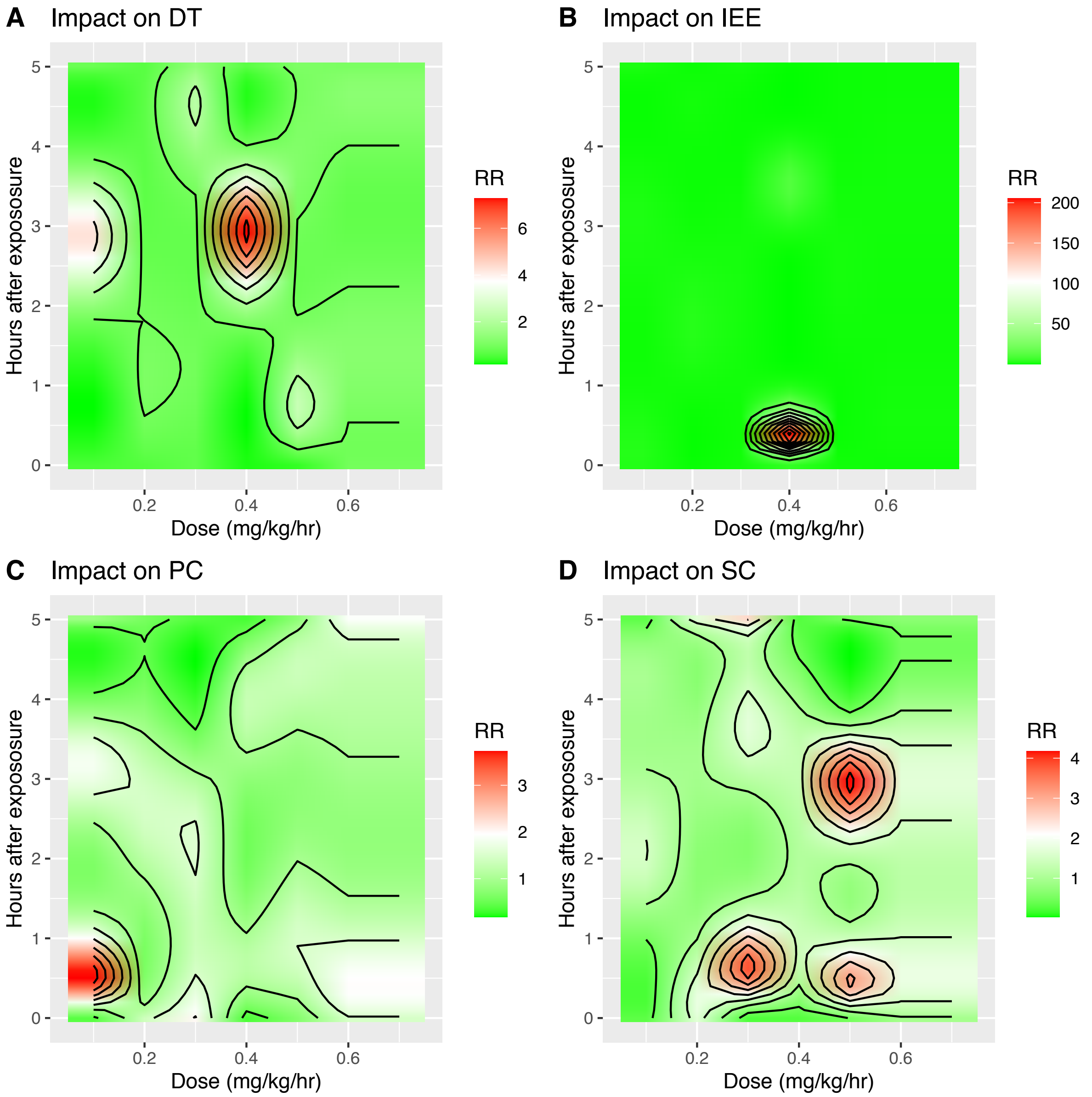

The midazolam was entered into the distributed lag non-linear model with two dimensions: dose and time lag. Exposure is considered as an instantaneous event that midazolam discontinues after relevant dose of exposure (x-axis). Other covariates including tidal volume, work of breathing, PEEP, plateau pressure, mode of ventilation and day hours were adjusted. The red color shows increased risk of asynchrony and the light green color shows the reduced risk of asynchrony. Note that small dose of midazolam increased the risk of IEE.

Abbreviations: IEE: ineffective effort; DT: double triggering; SC: short cycling; PC: prolonged cycling.

Figure S2. Impact of Sufentanil on four types of asynchrony.

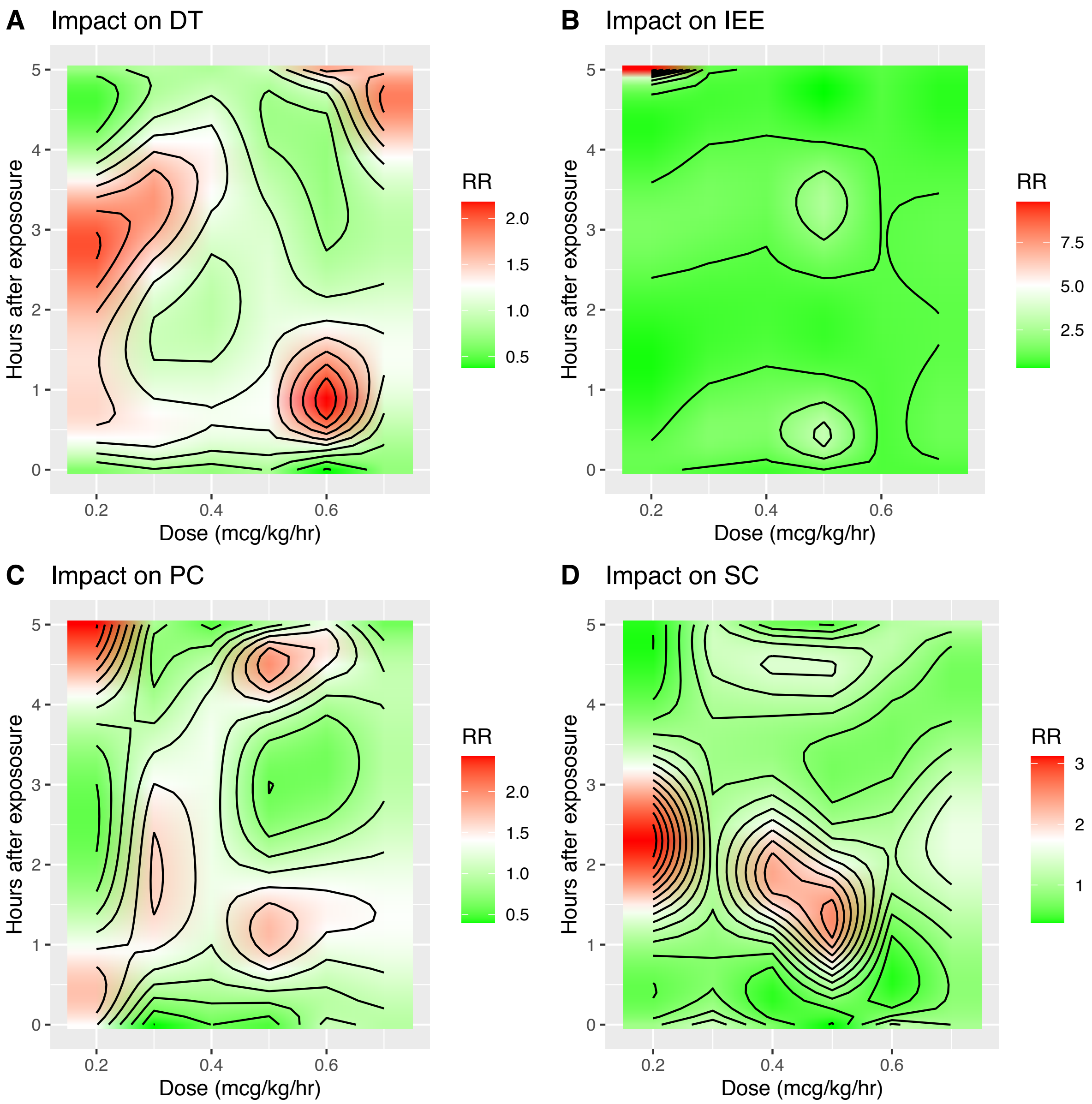

The sulfentanil was entered into the distributed lag non-linear model with two dimensions: dose and time lag. Exposure is considered as an instantaneous event that sulfentanil discontinues after relevant dose of exposure (x-axis). Other covariates including tidal volume, work of breathing, PEEP, plateau pressure, mode of ventilation and day hours were adjusted. The red color shows increased risk of asynchrony and the light green color shows the reduced risk of asynchrony. Note that sulfentanil was able to reduce the risk of IEE, PC, SC and DT shortly after infusion.

Abbreviations: IEE: ineffective effort; DT: double triggering; SC: short cycling; PC: prolonged cycling.
